# Spontaneous Bladder Rupture in a Catatonic Schizophrenia Patient

**DOI:** 10.1155/2023/4277372

**Published:** 2023-11-21

**Authors:** Megumi Miyakoshi, Takayuki Arai, Shin Kurose, Masataka Kaji, Jun Nakane, Mitsumoto Onaya, Akihiro Koreki

**Affiliations:** ^1^Department of Psychiatry, National Hospital Organisation Shimofusa Psychiatric Medical Centre, 578 Hetacho Midori-ku, Chiba 266-0007, Japan; ^2^Department of Urology, Chiba University Graduate School of Medicine, Chiba, Japan

## Abstract

Catatonia is a psychiatric emergency in schizophrenia that often leads to excessive activation of the sympathetic nervous system. Urinary retention in catatonia is often underestimated but has potentially detrimental consequences. Herein, we present the case of a woman in her 40s with schizophrenia treated for catatonia during a relapse. When treated as an inpatient, the patient suddenly complained of severe abdominal pain. Computed tomography revealed a spontaneous rupture of the posterior wall of the bladder, requiring emergency repair surgery in the urology department. The patient was readmitted to our hospital following surgery and ultimately discharged 1 month later. Bladder rupture is life-threatening, and delayed diagnosis and treatment can be fatal. This case report serves as a warning that psychiatrists should not overlook urinary retention in patients with catatonia and should consider bladder rupture in the differential diagnosis when these patients have abdominal pain.

## 1. Introduction

Catatonia is a psychiatric emergency in schizophrenia that often leads to severe comorbidities, such as pneumonia, pulmonary embolism, arrhythmia, and sepsis. Furthermore, the mortality rate is significantly higher for patients with catatonia than for those without [1]. Reports also suggest that catatonia-related comorbidities may be related to the excessive activation of the sympathetic nervous system [1, 2].

Urinary retention, a symptom frequently observed in cases of excessive sympathetic nervous system activation [3], is often underestimated but has potentially detrimental consequences, such as bladder rupture. Spontaneous rupture of the bladder is an extremely rare emergency that can be misdiagnosed due to its non-specific clinical presentation, leading to catastrophic complications. Spontaneous bladder rupture is usually a life-threatening condition. Bladder ruptures are classified as either traumatic or spontaneous. Only 3.4% of cases with bladder ruptures are spontaneous. The incidence of spontaneous bladder rupture is extremely low, approximately 1 in 50,000–126,000 [4]. Herein, we present a case of bladder rupture resulting from urinary retention in a patient with catatonia and subsequent early transfer to an emergency hospital for successful treatment.

## 2. Case Presentation

### 2.1. Patient Information

**Figure 1:**
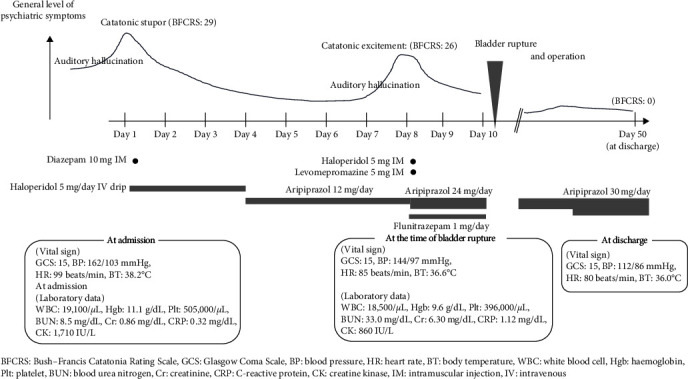
Clinical course of this case.

Figure 1 summarises the current clinical course of the patient. The patient was a woman in her 40s with schizophrenia. She had a history of a caesarean section but otherwise had no significant medical history. She had never been a regular user of either tobacco or alcohol. There was no family history of psychiatric illness. The patient started experiencing auditory-verbal hallucinations and delusions in her 20s and was treated with antipsychotics, which effectively controlled the symptoms for several years until the medications were discontinued, leading to a relapse and catatonia.

Upon admission, the patient presented with a catatonic stupor. An intramuscular injection (IM) of 10 mg of diazepam immediately resolved the stupor, and subsequent treatment for acute psychosis with haloperidol was administered via a continuous intravenous infusion of 5 mg/day for 3 days, improving her symptoms. She became communicative and was able to eat. Therefore, she was prescribed 12 mg/day of aripiprazole.

On the 8th hospitalisation day, the patient's symptoms deteriorated: she presented with auditory hallucinations and delusions, along with bizarre behaviours, such as walking with her eyes closed, staring and tweeting, and fluttering her hands. Her symptoms improved after the temporary use of haloperidol (5 mg IM) and levomepromazine (5 mg IM), an increased dose of aripiprazole (to 24 mg/day), and flunitrazepam administration (1 mg/day). On the 10th hospitalisation day, she suddenly complained of severe abdominal pain.

Regarding her physical condition during the initial course at our hospital, the patient had slight rhabdomyolysis (creatine kinase level: 1,710 IU/L (reference (ref.): 41–153 IU/L)) at admission. Her vital signs during the acute psychiatric phase were as follows: blood pressure: 162/103 mmHg, heart rate: ∼99 beats/min, and body temperature: ∼38.2°C. There was also no evidence of infection based on physical examination, blood tests, including procalcitonin, urine tests, and chest and abdominal computed tomography (CT). Polydipsia and reduced blood potassium levels were not observed throughout the clinical course. Additionally, nurses checked the patient's normal urine volume range until the third hospitalisation day and found that she had normal voiding frequency until the rupture event.

With the exception of a large amount of urine accumulated in the bladder, chest, and abdominal CT scans on the third hospitalisation day did not show any abnormalities.

From the patient's perspective, she was aware of the difficulty in voiding, slow stream, and overflow incontinence.

### 2.2. Clinical Findings

At the time of the sudden severe abdominal pain, the patient's vital signs were as follows: conscious level 15 according to the Glasgow coma scale [5], blood pressure: 144/97 mmHg, heart rate: 85 beats/min, and body temperature: 36.6°C. Physical examination revealed abdominal distension, negative guarding, but positive rebound tenderness. The relevant blood test results from the referring hospital were as follows: white blood cell count 18,500/*μ*L (ref.: 3,300–8,600*/μ*L), haemoglobin level 9.6 g/dL (ref.: 11.6–14.8 g/dL), platelet count 396,000*/μ*L (ref.: 158,000–348,000*/μ*L), blood urea nitrogen level 33 mg/dL (ref.: 8–20 mg/dL), creatinine level 6.3 mg/dL (ref.: 0.46–0.79 mg/dL), and C-reactive protein level 1.12 mg/dL (ref.: 0.00–0.14 mg/dL). Insertion of a urinary catheter revealed haematuria, and >2,000 mL of urine were drained.

### 2.3. Diagnostic Assessment

**Figure 2:**
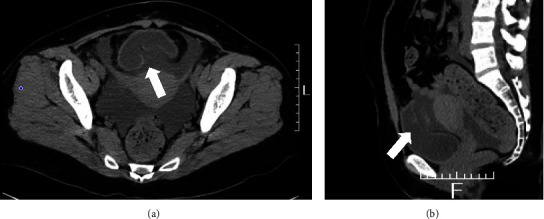
Computed tomography images of the bladder show a rupture from the posterior wall: (a) axial view; (b) sagittal view. Arrows point to the ruptured wall.

A CT scan, which was also performed at the referring hospital, revealed a rupture of the bladder (Figure 2). The radiological report described the thickness of the overall bladder wall, the absence of an expanding appearance of the bladder relative to its size, discontinuity of the posterior wall, and a marked volume of ascites. In addition, abdominal paracentesis revealed that the content of her ascites was similar to the results of her urine test. Since there was no history of an obvious traumatic event, she was diagnosed with spontaneous bladder rupture.

### 2.4. Therapeutic Intervention

Emergency repair surgery was performed in the urology department at Chiba University Hospital after an emergency transfer from our psychiatric hospital. An intra-abdominal approach through a median lower abdominal incision revealed a rupture that ran longitudinally down the posterior wall of the bladder, measuring approximately 5 cm. After trimming the defective tissue from the injured area, the bladder wall was sutured using two layers of 3–0 monofilament absorbable thread with intermittent sutures. No adhesion around the bladder was found. For standard infection control during the perioperative period, 3 g/day of sulbactam/ampicillin was administered for 3 days. As part of postoperative care, an indwelling urinary catheter was used to manage the patient's voiding.

### 2.5. Follow-Up and Outcomes of Interventions

Following surgery, the patient was readmitted to our hospital and ultimately discharged 1 month later after achieving stability with aripiprazole at a dosage of up to 30 mg/day. Her catatonia improved from 29 (at admission) and 26 (at the time of symptom deterioration) to 0 on the Bush–Francis Catatonia Rating Scale [6]. Her vital signs stabilised compared to those during the acute psychiatric phase: her blood pressure, heart rate, and body temperature after symptom improvement were 112/86 mmHg, 80 beats/min, and 36.0°C, respectively. This suggests that hyperactivation of the sympathetic nervous system due to catatonia was observed in her acute psychiatric phase. For follow-up after discharge, she had to visit both the psychiatry and urology departments at a hospital near her home. Considering her bladder condition, the risk of symptom exaggeration, and her preferences, we planned to maintain the catheter in place for continuous use, with scheduled monthly replacements.

## 3. Discussion

Bladder rupture is life-threatening, and delayed diagnosis and treatment can be fatal. We believe that early transportation from our psychiatric hospital to the emergency hospital contributed to successful treatment. Emergency blood tests and CT scans are usually unavailable in psychiatric hospitals, and psychiatrists need to evaluate the severity of abdominal pain through physical examination, the clinical course, and their expertise. As urinary retention in patients with catatonia is often underestimated, this case report can provide educational clinical findings about its detrimental consequences.

Various causes of spontaneous bladder rupture, including tumours, surgery, and alcohol consumption, have been observed, some of which include cases of schizophrenia and depression [4, 7]. Bladder rupture in patients with psychiatric disorders has been usually attributed to urinary retention due to psychotropic drugs [7]. For example, a case report described a woman with schizoaffective disorder who received treatment involving multiple psychotropic drugs, including four types of antipsychotic agents and one anticholinergic agent, leading to bladder rupture [8]. Additionally, there was a rare case of bladder rupture reported during electroconvulsive therapy in a patient with depression [9]. However, the cause of bladder rupture in our patient could be attributed to a specific condition characterised by catatonia.

Catatonia is accompanied by excessive sympathetic nervous system activation [1, 2], leading to urinary retention. In such cases, megacystis is also possible [2]. Our patient exhibited a slightly bimodal clinical course. At the time of admission, the patient developed a stupor, which immediately improved with benzodiazepine. The urinary volume was within the normal range, and a normal bladder was confirmed on CT on day 3, while she was aware of the difficulty in voiding, slow stream, and overflow incontinence. This suggests moderate urinary retention but not complete obstruction yet at that time. Her overflow incontinence could have hindered the expansion of the bladder. Therefore, we assume that the deterioration of the patient's symptoms on day 8 may have resulted in the exacerbation of urinary retention, finally leading to bladder rupture. Normal bladder capacity in adults' ranges from 300 to 400 mL, and the normal voiding frequency is approximately 8 times/day [10]. However, a previous study reported 1,800 mL of urine in the bladder in a case of catatonia due to severe urinary retention [11]. In our case, more than 2,000 mL of urine were in the bladder, and leaked urine in the intraperitoneal space was found when it ruptured. Although our patient's voiding frequency was within normal limits, her actual urine volume may have been underestimated. The patient's symptoms progressed from the intact bladder on day 3 (confirmed by CT) to symptom deterioration on day 8 and bladder rupture on day 10; thus, insufficient urine volume during this period could have rapidly increased the bladder wall pressure, causing the rupture.

In general, antipsychotics have a risk of urinary retention through various mechanisms [12], and drug effects cannot be completely ruled out in this patient. However, we do not believe that the medications were the primary causal factors for the patient presented here. This is because she was aware of the difficulty in voiding, slow stream, and overflow incontinence, suggesting that urinary retention developed before the use of antipsychotics. Therefore, it could be possible that these medications further contributed to the urinary retention, finally leading to the rupture. Beyond the pharmacological profiles of antipsychotic drugs, the safety and effectiveness of these antipsychotics in patients with catatonia remain inconclusive [13]. Nonetheless, the patient's “psychotic” symptoms improved with the use of these antipsychotics and flunitrazepam, and neuroleptic malignant syndrome was not induced. On the other hand, we speculate that these treatments were insufficient in deactivating her sympathetic nervous system. This suggests that early consideration of modified electroconvulsive therapy may be necessary in such catatonic cases [2]. As for other potential factors, obvious structural vulnerabilities were not identified. Furthermore, there was no adhesion around the bladder or other anomalies noted in the surgical records. There was also no evidence of polydipsia or trauma. There was no history of alcohol intoxication, substance overdose, and radiotherapy. Therefore, considering the specific nature of catatonia [2], we suspect that catatonia was the primary cause of the ruptured bladder. For the prevention and early intervention of severe comorbidity of patients with catatonia, it is crucial to implement strict monitoring, incorporating the patient's perspective and clinicians' professional prediction, and considering early modified electroconvulsive therapy administration.

This case report has a limitation of lacking structured evaluation because the patient was treated in a clinical practice, not as part of a clinical trial. However, we believe that our discussion, based on all available information, provides a valid explanation for her bladder rupture.

In conclusion, this case report serves as a warning that psychiatrists should not overlook urinary retention in patients with catatonia and should consider bladder rupture in the differential diagnosis when these patients have abdominal pain.

## Data Availability

The patient's details are anonymised and thus unavailable.
